# Dysgenetic Polycystic Disease of Minor Salivary Gland: A Rare Case Report and Review of the Literature

**DOI:** 10.1155/2017/5279025

**Published:** 2017-01-19

**Authors:** N. Srikant, Shweta Yellapurkar, Karen Boaz, Mohan Baliga, Nidhi Manaktala, Ankita Sharma, Shakthi Dorai, Prajwal Pai

**Affiliations:** ^1^Department of Oral Pathology and Microbiology, Manipal College of Dental Sciences, Mangalore, Manipal University, Manipal, India; ^2^Department of Oral Surgery, Manipal College of Dental Sciences, Mangalore, Manipal University, Manipal, India; ^3^Kasturba Medical College, Mangalore, Manipal University, Manipal, India

## Abstract

Polycystic (dysgenetic) disease of the salivary glands is a rare entity that has only recently been described in the literature. The disease is more commonly seen in females and majority of the cases have presented as bilateral parotid gland swellings. This case presenting in a 21-year-old male is the first of this unusual entity involving solely the minor salivary gland on the lower lip. This case report highlights the importance for the clinician to be aware of this differential diagnosis, when treating an innocuous lesion like a mucocele.

## 1. Introduction 

A diversity of neoplastic and nonneoplastic salivary gland lesions exists which present as cystic lesions. Salivary gland pathologies present with overlapping histopathological features making it a challenge for the histopathologist as well as the surgeon to diagnose and plan the treatment. Amongst all lesions causing enlargement of the salivary glands, nonneoplastic cysts account for approximately 6% of which one of the rarest cystic condition is dysgenetic polycystic disease (DPD) of salivary gland with distinctive histopathology [1]. It is one of the rare causes of parotid gland swelling, and only 18 cases are reported in the literature till date. In 1962, Mihalyka reported a case with possible features of DPD but the diagnosis could not be substantiated as the histologic appearance was not described [2]. Seifert et al. (1981) described the clinical and histologic characteristics of this disease and termed this entity as* bilateral dysgenetic polycystic parotid glands* [3].

In the current article, we describe a unique case of* dysgenetic polycystic disease of the lower lip *with review of the reported cases.

## 2. Case Report

A 21-year-old male patient reported to the Department of Oral and Maxillofacial Surgery with the chief complaint of swelling on the lower lip of 10 days' duration. The patient gave a history of an increase in swelling in the morning and before meals and the swelling usually subsided by evening. On general physical examination the patient was well built and nourished with no relevant past medical history. None of the family members of the patient had reported a similar lesion.

**Figure 1:**
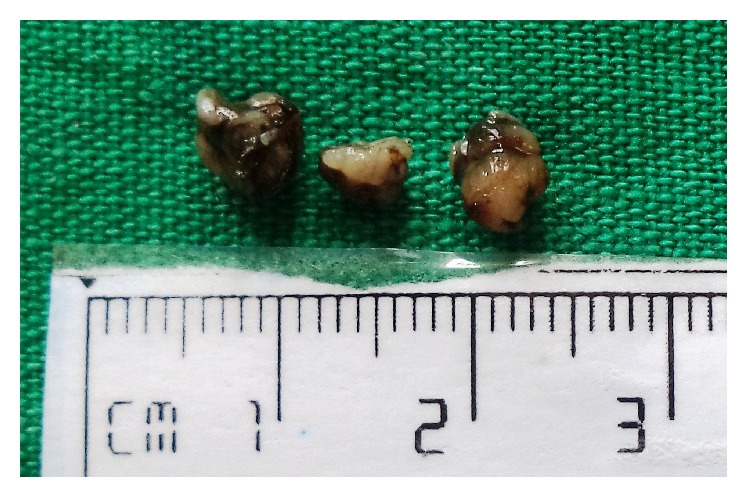
Gross appearance of the tissue.

On intraoral examination, a fluctuant swelling measuring approximately 1 × 1 cm in diameter was seen on the lower lip. On palpation, the swelling was nontender, mobile, and soft in consistency. Based on the clinical presentation, a provisional diagnosis of mucocele was rendered. The lesion was surgically excised under local anesthesia and sent for histopathological examination as three soft tissues, white to brown in colour, smooth to rough surface, fluctuant to firm in consistency, and collectively measuring approximately 1.4 × 1.2 × 0.4 cms (Figure 1).

**Figure 2:**
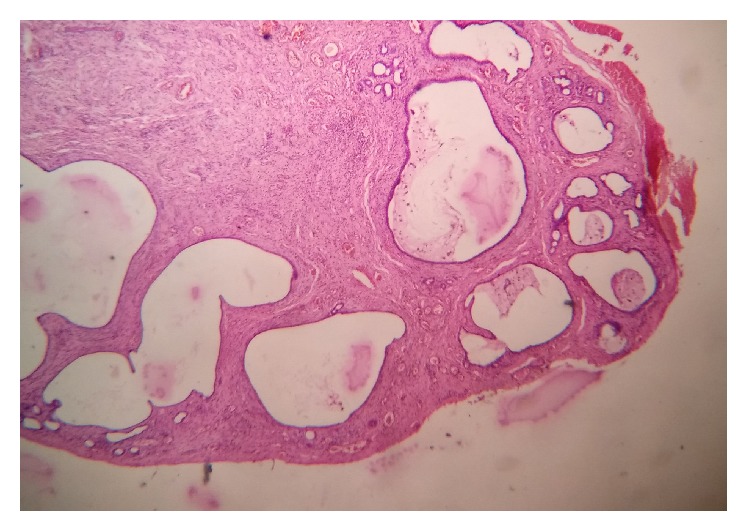
Multiple cystic spaces supported by a connective tissue stroma.

**Figure 3:**
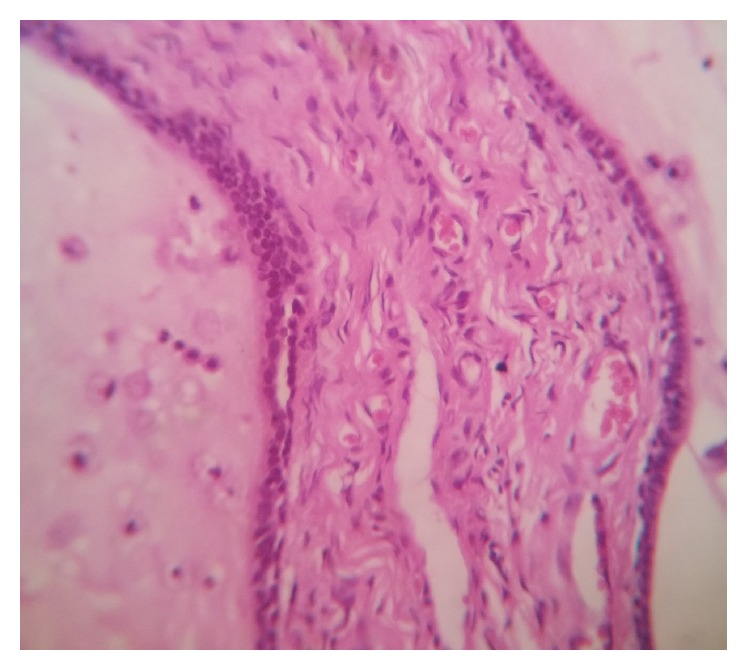
Cystic lumen lined by cuboidal/columnar cells with lumen containing mucin and muciphages.

Microscopic examination in a low power revealed the presence of numerous ectatic, variable-sized ducts interspersed in a fibrocellular connective tissue stroma (Figure 2). On higher magnification, the ductal/cystic spaces showed mucin pooling with abundant muciphages (Figure 3). Associated mucous minor salivary glands showed acinar atrophy with squamous metaplasia of the excretory duct. Areas of hemorrhage were also seen overlying the lesional tissue.

Considering the location and the benign clinical appearance of the lesion, the differential diagnosis was narrowed down to four possible lesions: namely, mucocele, lipoma, sclerosing polycystic adenosis, and polycystic dysgenetic disease in order of their frequency of occurrence. These can be easily distinguished histopathologically. Mucocele is unicystic in nature with watery spinnbarkeit nature of material in aspirate, lipoma presents as an encapsulated mass of mature adipocytes, and sclerosing polycystic adenosis exhibits fibrosis and hyperplastic ductal and acinar epithelial elements.* Thus, this classical histopathological picture aided in diagnosing the case as a “polycystic dysgenetic disease of minor salivary glands.”*

The patient underwent assessment of all glandular tissues in the whole body including other salivary glands, liver, kidney, and pancreas and no significant findings were elicited. The lesion in the lower lip healed uneventfully and a review at 18 months showed no recurrence.

## 3. Discussion 

Dysgenetic polycystic disease (DPD) is a rare benign cystic condition affecting the parotid glands with only 18 well-documented cases reported in the literature (Table 1). The lesion is typically limited to a salivary gland lobule and typically lacks any clinical and histologic signs of inflammation. The disease is thought to be a developmental disturbance of the distal duct system with no defects seen in the other ducts [3].

A systematic review of the literature revealed 18 documented cases of dysgenetic polycystic disease (DPD) of salivary glands. The mean age of diagnosis is 31.2 ± 22.28 years and median age is 23 years (range 6–83 years). The lesion is twice as common in females with the male to female ratio being 6 : 13. The lesion is predominantly seen in the parotid. 74% of the cases occurred in the parotid gland (9 cases were bilateral and 5 cases were unilateral on right side alone). One case showed bilateral submandibular gland involvement and one case showed involvement of bilateral parotid, submandibular, and minor salivary gland. The present case would be the 19th to be documented and the first to be occurring only in the minor salivary gland.

Certain clinical characteristics are deemed to be unique to this lesion when it presents in the major salivary glands. These include

- history of a recurrent, asymptomatic swelling of the involved gland and no association with any clinical abnormality of salivation or anomaly of salivary gland,
- presence of enlarged, nontender bilateral parotid swelling for months or years: Batsakis et al. (1988) described the histologic findings in three female patients with this disorder, two of which had bilateral parotid swelling since childhood, and one had unilateral swelling since puberty and subsequently developed swelling of the other gland during young adulthood [5]. Garcia et al. (1998) reported the first case affecting the submandibular gland bilaterally (French literature) [11],
- recurrent parotid swelling often occurring in childhood, while symptoms of this disorder might be delayed until adulthood [8].

## 4. Etiology and Pathogenesis

Many concepts have been proposed regarding the etiology of this condition.

- Genetic susceptibility: five of the cases reviewed gave a positive family history of similar lesion in mother, maternal grandmother, or father; however, in the present case there was no relevant family history. Smyth et al. (1993) reported this condition to be familial with mother and daughter diagnosed with DPD. Moreover, the female predilection exhibits sex linked inheritance [7]. Ficarra et al. (1996) reported DPD of parotid glands in a woman, her mother, and her maternal grandmother, thus suggesting an autosomal dominant inheritance [10]. However, Ashok Kumar et al. (2013) reported a case of DPD involving the salivary gland where the mother and sister of the patient had multiple odontogenic keratocyst (OKC) of the lower jaw [2]. The genetic association seen with DPD justifies the term “dysgenetic” for this lesion.
- Histologically the lesion shows multiple ductal ectasias resembling a mucous retention cyst. The presence of microliths/spheroliths histologically has been reported by Seifert et al. (1981), Batsakis et al. (1988), Brown et al. (1995), and Layfield and Gopez (2002) [3, 5, 8, 12]. The presence of multiple sialoliths may lead to mucous retention phenomenon presenting with a “polycystic” nature histologically.
- Hormonal influence is also considered to exacerbate the underlying condition. Brown et al. (1995) reported a bilateral parotid swelling in a 31-year-old, 4-month pregnant woman which regressed within 4–6 months after parturition establishing a hormonal influence [8].
- Alternative theory includes the developmental malformation of the intercalated duct system [3]. It has been compared with the developmental process resulting in cystic malformations of the pancreas, lung, kidney, and liver [1]. No evidence of cystic formation occurring outside the parotid has been documented in cases in the literature. Disturbance in the ramification and canalization of the ductal system during the second stage of the development of salivary gland that extends to the end of the seventh embryonal month might lead to formation of DPD. In the present case, the investigations did not reveal the presence of any such cystic lesions elsewhere in the patient.

## 5. Histopathological Features

Grossly, the cut surface of such lesions usually presents with an exaggerated lobularity and yellow to ivory nodules with a spongy consistency [6]. These nodules histologically represent the ectatic ducts. The glandular architecture is preserved but the lobules are distended and replaced by epithelium-lined cysts exhibiting a honeycombed or lattice-like appearance. Amidst the cysts, small, variable-sized residual islands of glandular acini are present. These cystic spaces are variably lined by flattened cuboidal or columnar epithelium. The columnar cells contain abundant eosinophilic cytoplasm and rounded luminal borders resembling apocrine cells. Few areas show bud-like epithelial proliferations and in others spur-like incomplete septae are seen. Several epithelial cells contain prominent cytoplasmic vacuoles with lipid material. Sometimes ducts open directly into cysts and some acinar units communicate with the cyst suggesting its origin from intercalated ducts. There is flocculent, eosinophilic material and a few scattered macrophages found in most of the cystic lumina. Along with this many cysts contain eosinophilic bodies with concentric and radial patterns that resemble spheroliths and microliths that have been reported in 6 cases [3, 5, 12].

Brown et al. (1994) reported seeing amyloid-like material on aspiration which showed positivity with Congo red stain. However, an open biopsy of the same case showed the amyloid-like material to represent eosinophilic spheroliths [8]. These findings were similar to those of Dobson and Ellis who described such spheroliths to be congophilic and consistent with amyloid [4].

## 6. Treatment

As the lesion is microscopically benign, the current choice of treatment is complete excision. Cases that have involved the parotid gland have been successfully treated by lobectomy or superficial parotidectomy. As multiple salivary glands may be involved, examination of the other glandular structures like the other salivary glands, pancreas, kidneys, spleen, and so forth, must be carried out. However, in view of the paucity of reported cases, long-term follow-up is essential to eliminate the possibility of recurrence and to screen for the potential involvement of other salivary glands [4]. Genetic testing of reported cases may highlight any mutation that results in such cases and also help in comparing with polycystic disease affecting other organ systems. In view of the potential for bilateral involvement of glands that may necessitate lobectomy as the preferred choice of treatment, salivary gland function may be compromised and thereby affect oral health.

## Figures and Tables

**Table 1:** Review of cases of dysgenetic polycystic disease reported in the literature.

S. number	Author (year)	Age (years)/gender	Site	Family history	Other findings
(1)	Seifert et al. (1981) [3]	6/female	Bilateral parotid	Father	Spheroliths
(2)	Seifert et al. (1981) [3]	65/female	Unilateral parotid		
(3)	Dobson and Ellis (1987) [4]	23/female	Bilateral parotid	Nil	Amyloid-like material
(4)	Batsakis et al. (1988) [5]	26/female	Bilateral parotid		Microliths/spheroliths
(5)	Batsakis et al. (1988) [5]	16/female	Bilateral parotid		Microliths/spheroliths
(6)	Batsakis et al. (1988) [5]	32/female	Bilateral parotid		Microliths/spheroliths
(7)	Warnock et al. [6]	2 cases (1 male and 1 female)			
(8)	Smyth et al. (1993) [7]	18/female	Bilateral parotid	Mother	
(9)	Brown et al. (1995) [8]	31/female	Bilateral parotid	Nil	Amyloid-like material in FNA. Lesion regressed after pregnancy
(10)	Ortiz-Hidalgo et al. (1995) [9]	65/female	Unilateral parotid	—	—
(11)	Ficarra et al. (1996) [10]	32/female	Bilateral parotid	Maternal grandmother and mother	
(12)	Garcias et al. (1998) [11]	Male	Bilateral submandibular		
(13)	Layfield and Gopez (2002) [12]	21/male	Bilateral parotid	Father	Spheroliths
(14)	Eley et al. (2011) [1]	8/male	Right parotid	NIL	
(15)	Ashok Kumar et al. (2013) [2]	21/female	Right parotid		Mother and elder sister had multiple odontogenic keratocysts
(16)	Guledgud et al. (2014) [13]	83/male	Right parotid	Nil	No recurrence
(17)	Koudounarakis et al. (2016) [14]	Female	Bilateral parotid, submandibular, and minor salivary gland		Bilateral parotid warthin's tumour, nasal polyps
(18)	Present case	21/male	Minor salivary gland	Nil	
